# Sphingosine-1-phosphate alleviates colitis by regulating macrophage polarization and PI3k-Akt signaling

**DOI:** 10.3389/fimmu.2025.1622094

**Published:** 2025-07-21

**Authors:** Liang Hu, Zi Yang, Ying Zhang, Conglin Du, Yang Yang, Zhichao Chang, Xiangchun Li, Zhaochen Shan

**Affiliations:** ^1^ Outpatient Department of Oral and Maxillofacial Surgery, Beijing Stomatological Hospital, Capital Medical University, Beijing, China; ^2^ Salivary Gland Disease Center and Beijing Key Laboratory of Tooth Regeneration and Function Reconstruction, School of Stomatology and Beijing Laboratory of Oral Health, Capital Medical University, Beijing, China; ^3^ Department of Endodontics, Beijing Stomatological hospital, Capital Medical University, Beijing, China; ^4^ Department of Oral and Maxillofacial and Head and Neck Oncology, Capital Medical University School of Stomatology, Beijing Stomatological Hospital, Capital Medical University, Beijing, China; ^5^ Department of Stomatology, The First Hospital of Qinhuangdao, Hebei, China

**Keywords:** sphingosine-1-phosphate, inflammatory bowel disease, intestinal epithelial integrity, macrophage, PI3K-Akt signaling pathway

## Abstract

**Introduction:**

Inflammatory bowel disease (IBD) is a complex disease that is characterized by tight junction loss and dysregulation of immune homeostasis. The repair of intestinal integrity and immune function in IBD remains a clinical challenge. Sphingosine-1-phosphate (S1P) has been reported to alleviate radiation-induced salivary gland damage by maintaining epithelial integrity. However, its potential to restore function during IBD has not yet been investigated.

**Methods:**

Dextran sulfate sodium (DSS) was added to the drinking water of C57BL/6 mice for 5 days to induce colitis. Subsequently, S1P and vehicle were injected intravenously on days 1, 3, and 5. Body weight, the disease activity index (DAI), and the histological activity index (HAI) were recorded. The level of apoptosis and expression of tight junction proteins among the groups were compared. We explored the underlying mechanisms of S1P using RNA sequencing.

**Results:**

S1P alleviated DSS-induced colitis by suppressing inflammatory cell infiltration, reducing ulcers, and maintaining intestinal epithelial junction integrity by increasing E-cadherin and occludin expression. S1P decreased apoptosis, suppressed M1 macrophage polarization and promoted M2 macrophage polarizaion. RNA sequencing revealed upregulation of the phosphatidylinositol 3-kinase/protein kinase B (PI3K-Akt) and chemokine signaling pathways in the DSS group compared with those in the S1P group.

**Conclusions:**

S1P alleviated colitis by maintaing the intestinal epithelial integrity, promoting the polarization of M2 macrophage, suppressing chemokines, and regulating PI3K/Akt signaling pathway.

## 1 Introduction

The incidence of inflammatory bowel disease (IBD) is increasing, with over 1 million people affected in the USA and ~ 2.5 million in Europe, resulting in substantial increases in healthcare costs (1). The etiology of IBD is complex and includes genetic factors, the intestinal microbiome, inappropriate diet, and immune responses (2, 3). Treating IBDs is complicated owing to their multidimensional etiology (3, 4). Ceramide is enzymatically converted to sphingosine, which is then phosphorylated by sphingosine kinase (Sphk) to form sphingosine-1-phosphate (S1P) (5). Sphingosine-1-phosphate (S1P) biosynthesis occurs through enzymatic conversion of ceramide to sphingosine, followed by sphingosine kinase (Sphk)-mediated phosphorylation (5). S1P is transported extracellularly via specific transporter and binds five S1P receptors (S1PRs) on cell membranes (6). Plasma S1P levels are elevated in ulcerative colitis (UC) patients compared to healthy individuals (7), highlighting its close connection of IBD.

The stability of the endothelial cell (EC) barrier is maintained via the S1P/S1P_1_ axis by remodeling focal adhesions (8). Moreover, the amounts of surface molecular markers of cell adhesion and cell-cell contact, such as platelet-endothelial cell adhesion molecule (PECAM)-1 and vascular endothelial (VE)-cadherin, are diminished when S1P_1_ expression is downregulated long-term (9). In addition, S1P can rapidly and continuously enhance cytoskeletal rearrangement by upregulating polymerized F-actin and cytochalasin B expression (10, 11).

Leukocyte trafficking and the consequential accumulation of pro-inflammatory mediators activate and perpetuate intestinal immune responses during IBD (12, 13). The role of S1P-S1PR signaling in immune trafficking and activation is critical in maintaining homeostasis and in pathological conditions associated with inflammatory diseases (12, 14). The daily oral administration of etrasimod (S1PR1/R4/R5 modulator) alleviates the colonic inflammatory response by downregulating the expression of T cell and monocyte markers in a cluster of differentiated CD4^+^CD45RB^high^ T cell transfer mouse model of colitis (15). Immune homeostasis in patients with IBD can be regulated by S1P by reducing the amount of circulating leukocytes trafficking into the colon (16).

Dextran sulfate sodium (DSS)-induced colitis animal models are commonly used to study IBD (17). Loss of tight junction protein expression occurs on day 1 after DSS administration, and pro-inflammatory cytokine expression increases (18, 19). The major symptoms and pathological changes that appear on day 5 after DSS administration include weight loss, severe rectal bleeding, reduced mobility, and abundant neutrophil infiltration into the lamina propria (20). The clinical symptoms of IBD can be alleviated by combining several pharmacological agents and nutritional approaches, but it is currently incurable (21). Previously, we found that injection of intra-submandibular glands with S1P resulted in the maintenance of homeostasis in the epithelial structure through the S1pr1/eNOS axis (22), which plays a vital role in the pathogenesis of colitis.

Therefore, in the present study, we aimed to determine the effects of S1P on IBD and the potential underlying mechanisms. We used DSS-induced colitis mice as animal models of IBD. The effects of S1P treatment were analyzed, and the underlying mechanisms were investigated. Our findings reveal the effects and underlying mechanism of interactions between S1P and the host immune system in intestinal mucosa, providing potential strategies for improving the treatment of IBD.

## 2 Materials and methods

### 2.1 Animal models

Forty 8-week-old male C57BL/6 mice were weighed, marked, and randomly assigned to the following groups (n = 10 per group): normal group (NT, sterile water), DSS group (3% w/v DSS for 5 days), S1P group (3% w/v DSS for 5 days, with intravenous (i.v.) injection of S1P on days 1, 3, and 5), and vehicle group (3% w/v DSS for 5 days, with vehicle i.v. injection on days 1, 3, and 5). The mice were maintained under specific pathogen-free (SPF) conditions at the Capital Medical University animal facilities, at ~ 22.2 ± 1.1°C with 30%–70% humidity. The Animal Care and Use Committee at Capital Medical University approved the study protocol (Approval ID: AEEI-2022-263).

The disease activity index (DAI) was scored based on total body weight (BW) loss, rectal bleeding, and stool consistency (23, 24). Weight loss was scored as 0 (< 2%), 1 (2%–5%), 2 (5%–10%), 3 (10%–15%), or 4 (≥ 15%). Rectal bleeding was scored as 0, normal; 1, brown; 2, reddish; and 3, bloody. Stool consistency was scored as 0, normal; 1, mildly soft; 2, very soft; and 3, watery (23).

### 2.2 Ethics statement

The animal study was approved by the Ethics Review Commission of the Laboratory Animal Center of Capital Medical University. The study was conducted in accordance with the local legislation and institutional requirements.

### 2.3 Drugs and reagents

We dissolved 30 g of DSS (9011-18-1; MP Biomedicals, Burlingame, CA, USA) in 1 L water to 3% (w/v) on the day of administration and dissolved 8 μg of S1P (HY-108496; MedChemExpress [MCE], Monmouth Junction, NJ, USA) in 100 μL of phosphate-buffered saline/1% bovine serum albumin (BSA). All mice were injected i.v. with 8 μg/20g.

### 2.4 Routine blood detection

The mice were anesthetized with pentobarbital sodium (50 mg/kg), and then blood was collected from the ophthalmic veins, and the platelet (PLT), white (WBC), and red (RBC) blood cell counts and hemoglobin (HGB) concentration were routinely determined.

### 2.5 Histological and immunofluorescence staining

Samples collected on day 5 were gently washed in PBS, cut into 1-cm sections, and fixed in 4% paraformaldehyde for 48 h. Tissues were coated in wax, sliced into 5-μm sections, then dewaxed using xylene. Then, the sections were histologically stained with hematoxylin and eosin (HE). The histological activity index (HAI) was calculated based on the histology of colonic epithelial damage and inflammatory cell infiltration (23, 24) and scored as 0, normal; 1, mild inflammation surrounding the intestinal fossa; 2, moderate inflammation in the mucosal muscular layer; 3, severe inflammation in the mucosal muscular layer with hemorrhage, and 4, severe inflammation in the submucosal layer.

Sections were dewaxed in xylene and dehydrated with an alcohol gradient. Antigens were retrieved in EDTA antigen repair buffer, and non-specific binding was blocked using 3% BSA. The sections were incubated overnight with primary antibodies against E-cadherin (1:100, GB12083-100, Servicebio, Wuhan, China), anti-F4/80 (GB113373, Servicebio), anti-CD86 (GB115630, Servicebio), and anti-CD206 (GB113497, Servicebio), followed by secondary antibodies.

### 2.6 Western blotting

The concentrations of proteins extracted from tissues stored at -80°C were determined using the Bradford method (Bio-Rad Laboratories, Hercules, CA, USA). Targeted proteins were incubated overnight with the following primary antibodies: 1:1000-diluted E-cadherin (A20798; ABclonal Technology, Woburn, MA, USA), 1:5000-diluted occludin (27260-1-AP; Proteintech Group Inc., Rosemont, IL, USA), 1:1000-diluted caspase3/cleaved caspase3 (14220, Cell Signaling Technology [CST], Danvers, MA, USA) and 1:1000-diluted BAX (14796; CST), Akt (1:1000, 60203-2-Ig; Proteintech), p-Akt (1:1000, 13038; CST), CCL2 (1:1000, 26161-1-AP; Proteintech), and GSK3β (1:1000, bs-0023M; Bioss, Beijing, China). Then, the proteins were incubated with 1:10,000-diluted secondary goat anti-rabbit or anti-mouse IgG (ab97051 and ab97040; Abcam, Cambridge, UK) for 1 h at room temperature. Protein concentrations were measured three times.

### 2.7 RNA sequencing

Total RNA extracted from colonic tissues and RNA libraries was sequenced (OE Biotech, Inc., Shanghai, China). The quality of the library was determined for bioinformatic assessment using principal component analysis (PCA) and statistical histograms of differentially expressed genes (DEGs). Common and specific DEGs among the groups were visualized using volcano maps. We assessed the upregulated and downregulated expression of DEGs and compared the top 20 terms of Kyoto Encyclopedia of Genes and Genomes (KEGG) enrichment analysis between the DSS and S1P groups. Potential associations between proteins were assessed using protein-protein interaction network (PPI) analysis.

### 2.8 Quantitative reverse transcription-PCR

We evaluated the gene expression of *Orm2, Cxcl2, Cxcl3, Ccl3*, and *Spp1* in colonic tissues. We extracted total RNA using an isolation kit (Vazyme, Nanjing, China) and transcribed it using a cDNA synthesis kit (Novoprotein, Suzhou, China). Specific RNA targets in samples were amplified using qRT-PCR with the primers shown in **Supplementary Table 1**, and the RNA targets were quantified using SYBR green. Transcript gene expression was normalized to that of Actb.

### 2.9 Cytokine enzyme-linked immunosorbent assay (ELISA)

Fresh colon samples (100 mg) were homogenized in 500 μL double-distilled water, and 200 μL portions of the homogenate were diluted 10-fold. The concentrations of the following cytokines were determined using ELISA kits as described by the manufacturers: IL-13 (Mouse IL-13 PI539, Beyotime Biotechnology, Haimen, China), IL-4 (Mouse IL-4; HJ179), IFN-γ (Mouse IFN-γ; HJ170), and TNF-α (Mouse TNF-α; HJ207; all from Epizyme Inc., Cambridge, MA, USA). The concentrations of GM-CSF and CCL2 were determined in 2-fold dilutions of homogenate stock solution using biotechne^®^ Mouse GM-CSF (MGM00) and Mouse CCL2/JE/MCP-1 (MJE00B) Quantikine ELISA kits (R&D Systems, Minneapolis, MN, USA).

### 2.10 Statistical analysis

All data and graphs were analyzed using GraphPad Prism version X9 (GraphPad Software Inc., La Jolla, CA, USA). Data were analyzed using one- or two-way ANOVA, followed by Tukey’s multiple comparisons or unpaired Student’s t-tests. Data are presented as means ± standard error of the means (SEM). Values with p < 0.05 were considered statistically significant.

## 3 Results

### 3.1 Colitis in DSS-induced mouse models was alleviated by S1P

**Figure 1:**
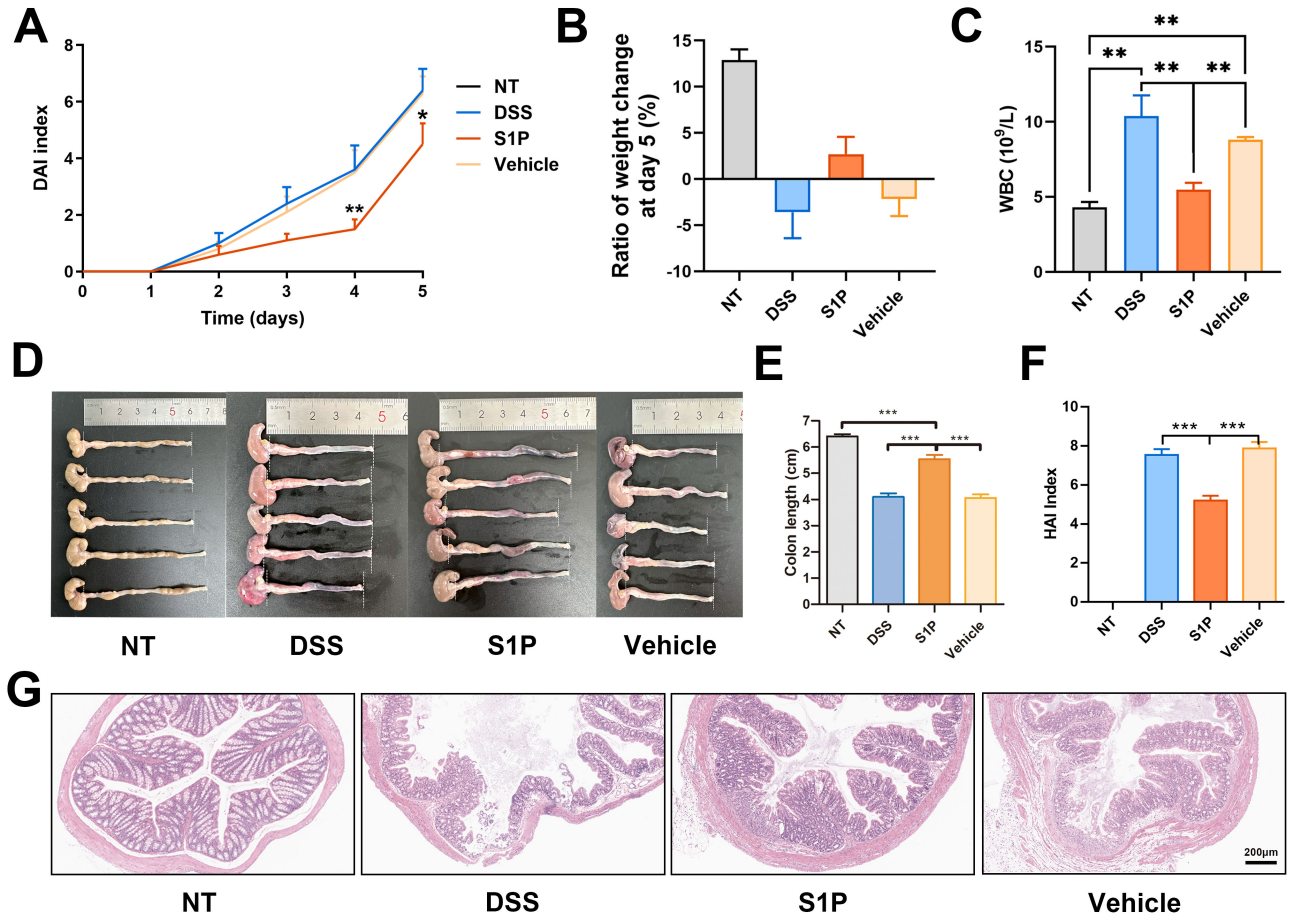
Colitis induced in mouse models DSS was alleviated by S1P. The DAIs (disease activity index) gradually increased after DSS administration. Compared at the same time point, the index was lower in S1P treated group. n=6, *p<0.05, **p<0.01 vs. DSS and Vehicle groups **(A)**. The body weight was decreased at day 5 in DSS and Vehicle group, and slightly increased in S1P group **(B)**. The WBC level at day 5 were checked, and was significantly increased by DSS. In S1P group, the WBC level was decreased. n=6, **p<0.01 **(C)**. The colon of experimental animals was photographed (n=5) and the length was measured. Both in DSS and Vehicle group, the colonic length was significantly deceased, and partially recovered in S1P group. n=6, ***p<0.001 **(D, E)**. The HAIs (histological activity index) was measured based on histological observation and HE staining was showed. The HAIs was significantly higher in DSS and Vehicle group compared with that in S1P group. n=8, ***p<0.001 **(F, G)**.

The colons of the DSS-induced colitis mouse models were significantly damaged (**Supplementary Figure 1A**). We measured changes in BW from day 1 to day 5 (**Supplementary Figure 1B**). The DAIs were significantly lower in the S1P group compared with those of the DSS group, indicating that S1P suppressed clinical symptoms in the models (**Figure 1A**). The BW of mice on day 5 was reduced in the DSS and control groups but slightly increased in the S1P group (**Figure 1B**). The number of WBCs in the peripheral blood was significantly increased in the DSS and vehicle groups and reduced in the S1P group (**Figure 1C**), indicating that systemic inflammation was improved in the S1P group. PLT, RBC counts, and HGB levels were reduced in the DSS and vehicle groups compared with those in the NT group and were slightly restored in the S1P group (**Supplementary Figures 1C–E**). Intravenous S1P administration alleviated pathogenic changes in the colon, such as hyperemia, swelling, and reduced length induced by DSS (**Figures 1D, E**). The results of the HAI analysis revealed that S1P mitigated histopathological changes in the colon (**Figure 1F**). Severe inflammation was accompanied by various degrees of ulceration in the mucosal muscular and submucosal layers in the DSS and vehicle groups. Inflammatory infiltration and ulceration were suppressed in the S1P group (**Figure 1G**).

### 3.2 Colitis induced by DSS was alleviated by S1P through maintaining intestinal epithelial junction integrity

**Figure 2:**
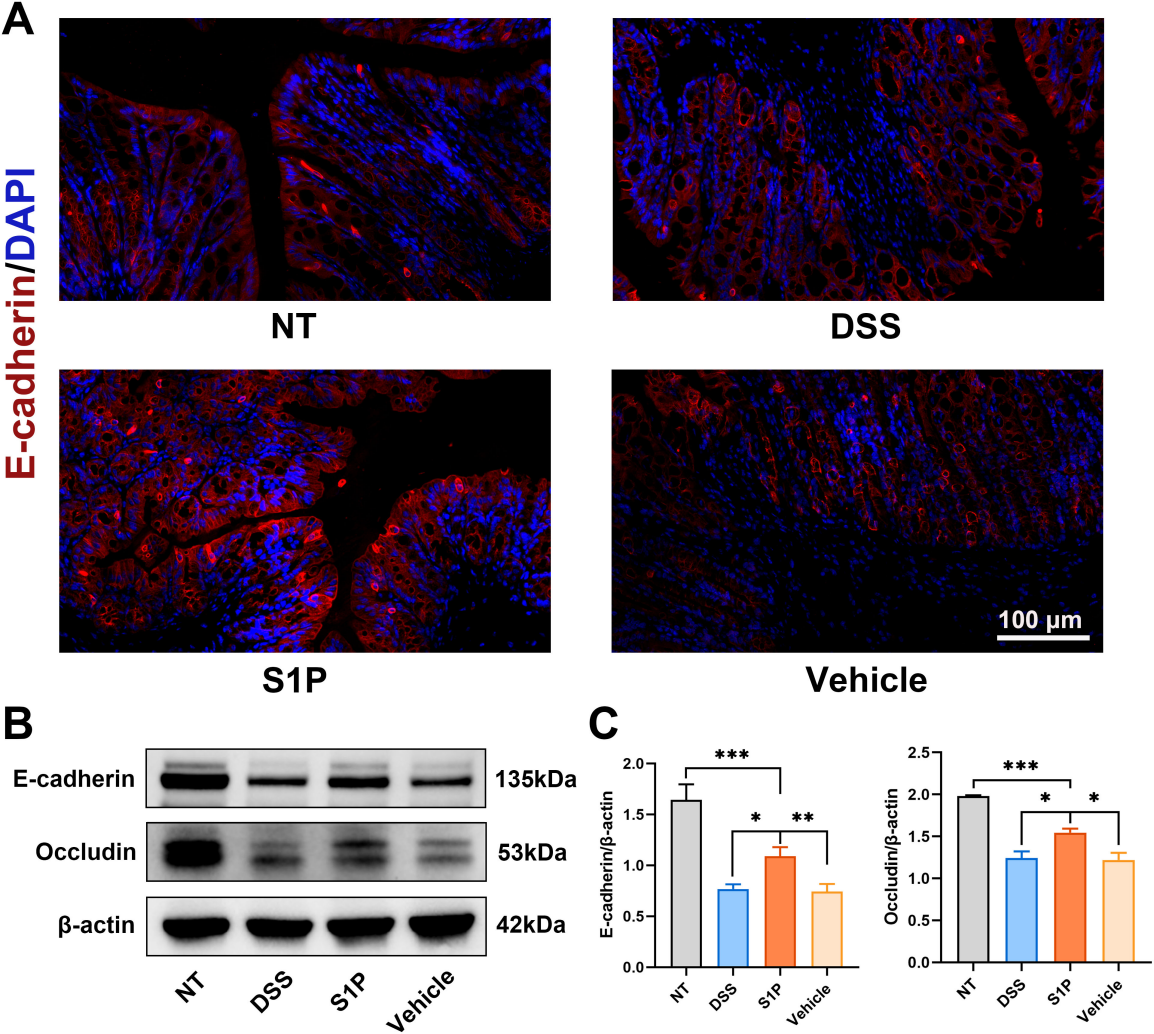
Colitis induced by DSS was alleviated by S1P through maintaining intestinal epithelial junction integrity. Tight junction protein, E-cadherin was detected by IF and results showed that the expression was downregulated by DSS and partially recovered in S1P group. n=8 **(A)**. The protein level of E-cadherin and Occludin detected by WB showed that DSS administration could significantly decrease the expression of tight junction protein, and S1P could partially recover the expression. n=3, *p<0.05, **p<0.01, ***p<0.001 **(B, C)**.

Colitis induced by DSS impairs intestinal barrier function by downregulating tight junction protein expression (25). E-cadherin is crucial for regulating colon homeostasis and its expression is downregulated in the colonic mucosa of patients with IBD (26). Our results revealed that S1P reversed the decrease in E-cadherin expression in the DSS and vehicle groups compared with that in the NT group (**Figure 2A**). Western blotting showed that the levels of E-cadherin and occludin were upregulated by S1P compared with the DSS group (**Figures 2B, C**). These findings confirmed that S1P maintained the integrity of the intestinal epithelial junction.

### 3.3 Apoptosis rates in the colonic epithelium of mice induced by DSS were decreased by S1P

**Figure 3:**
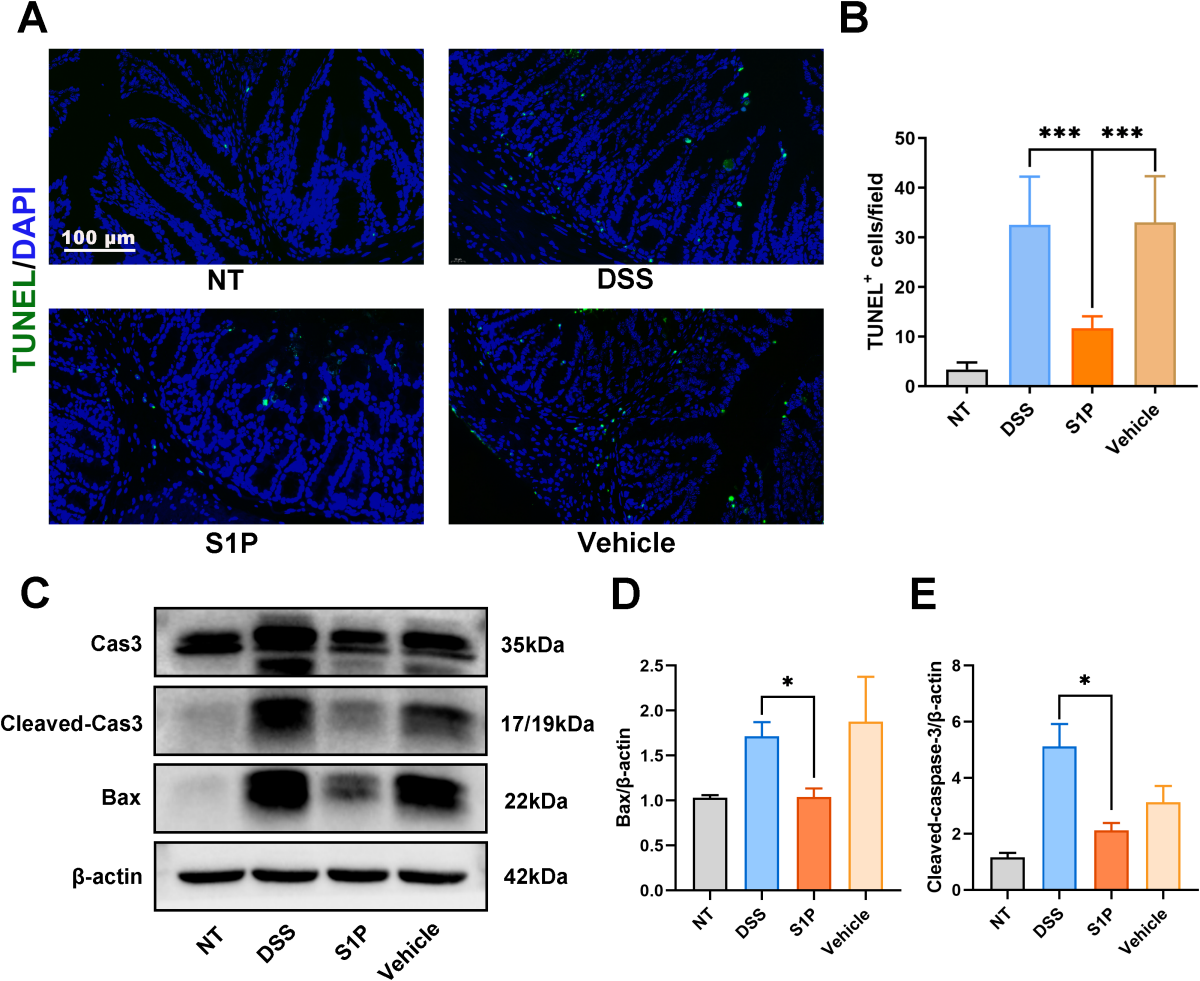
Apoptosis rates in the colonic epithelium induced by DSS were decreased by S1P. TUNEL staining showed that the positive apoptotic cells per field was increased in DSS and Vehicle group, and decreased in S1P group. n=8, ***p<0.001 **(A, B)**. The protein level Cleaved Cas3/Cas 3 and BAX were upregulated in DSS and Vehicle group, and downregulated in S1P group. n=3, *p<0.05 **(C–E)**.

We determined the level of apoptosis in colonic tissues using terminal deoxynucleotidyl transferase dUTP nick end labeling (TUNEL) assays. We observed significantly more TUNEL-positive cells per field in the DSS and vehicle groups and less in the S1P group compared with those in the NT group (**Figures 3A, B**). The levels of the apoptosis-related markers cleaved caspase3 and Bcl-2-associated X (Bax) protein were increased in the DSS and vehicle groups and decreased in the S1P group (**Figures 3C–E**). These results indicated that S1P suppressed DSS-induced apoptosis in the colonic epithelium.

### 3.4 Chemokine and PI3K-AKT signaling were upregulated in the DSS group compared with that in the S1P group

**Figure 4:**
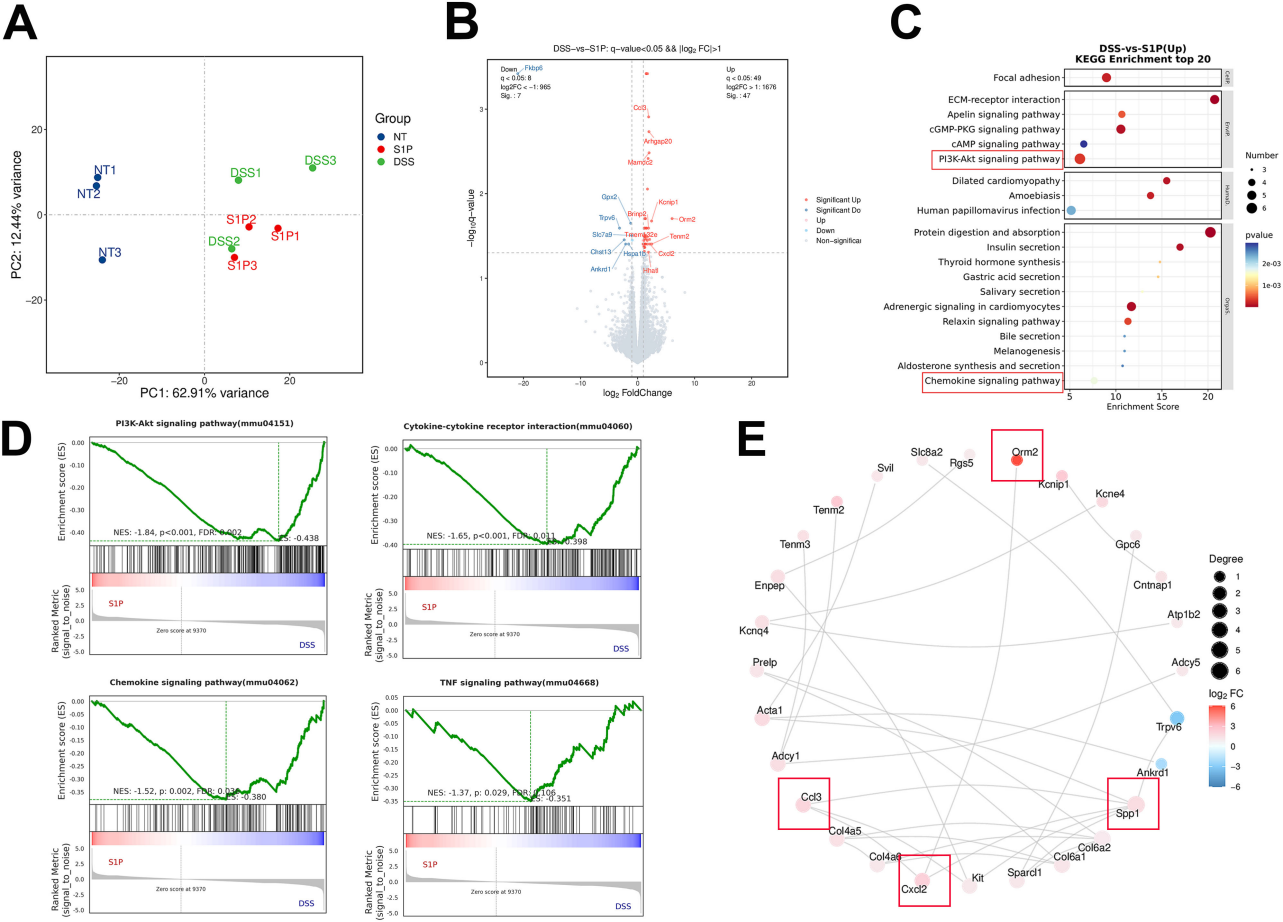
Chemokine and PI3K-AKT signaling was upregulated in DSS compared with S1P group. PCA results showed that samples from these three groups were distinct from each other **(A)**. Differentially expressed volcano map and differential gene grouping cluster map were presented between DSS and S1P group (**B, C** up: red, down: blue). KEGG analysis showed that compared with DSS group, PI3K-Akt signaling pathway, Chemokine signaling and cytokine-cytokine receptor interaction were upregulated in DSS group compared with that in S1P group **(D–G)**. PPI analysis of associated top 28 proteins. Red represents up-regulated differentially expressed genes, while blue represents down-regulated differentially expressed genes **(H)**. n=3 for RNA Sequencing.

We confirmed that S1P alleviated DSS-induced colitis in mice by maintaining intestinal epithelial junction integrity and inhibiting apoptosis. Subsequently, we explored possible mechanisms underlying the therapeutic effects of S1P. We sequenced RNA from colonic tissues obtained from the NT, DSS, and S1P groups. Clustering analysis revealed that the gene profiles in these samples from these groups were distinct from each other (**Figure 4A**). We compared common and specific DEGs between the DSS and S1P groups (**Supplementary Figure 2A**) and 47 upregulated and 7 downregulated genes (q < 0.05, **Supplementary Figure 2B**). Further analysis of the sequencing data revealed that *Fkbp6, Gpx2, Trpv6, Slc7a9, Chst13, Hspa1b*, and *Ankrd1* levels were significantly upregulated and *Ccl3, Arhgap20, Mamdc2, Kcnip1, Brinp2, Orm2, Tenm2, Cxcl2, Tmem132e*, and *Hhatl* levels were significantly downregulated in the S1P group compared with those in the DSS group (**Figure 4B**). The results of the KEGG analysis revealed downregulated focal adhesion, ECM-receptor interaction, PI3K-Akt and chemokine signaling in the S1P group compared with that in the DSS group (**Figure 4C**). The levels of DEGs associated with the PI3K-Akt signaling pathway differed between the DSS and S1P (**Figure 4D**). PPI analysis revealed associations among the top 28 proteins (**Figure 4E**). The RNA sequencing data revealed the involvement of larger loci with more closely related genes.

### 3.5 S1P regulated PI3K-Akt and chemokine signaling

**Figure 5:**
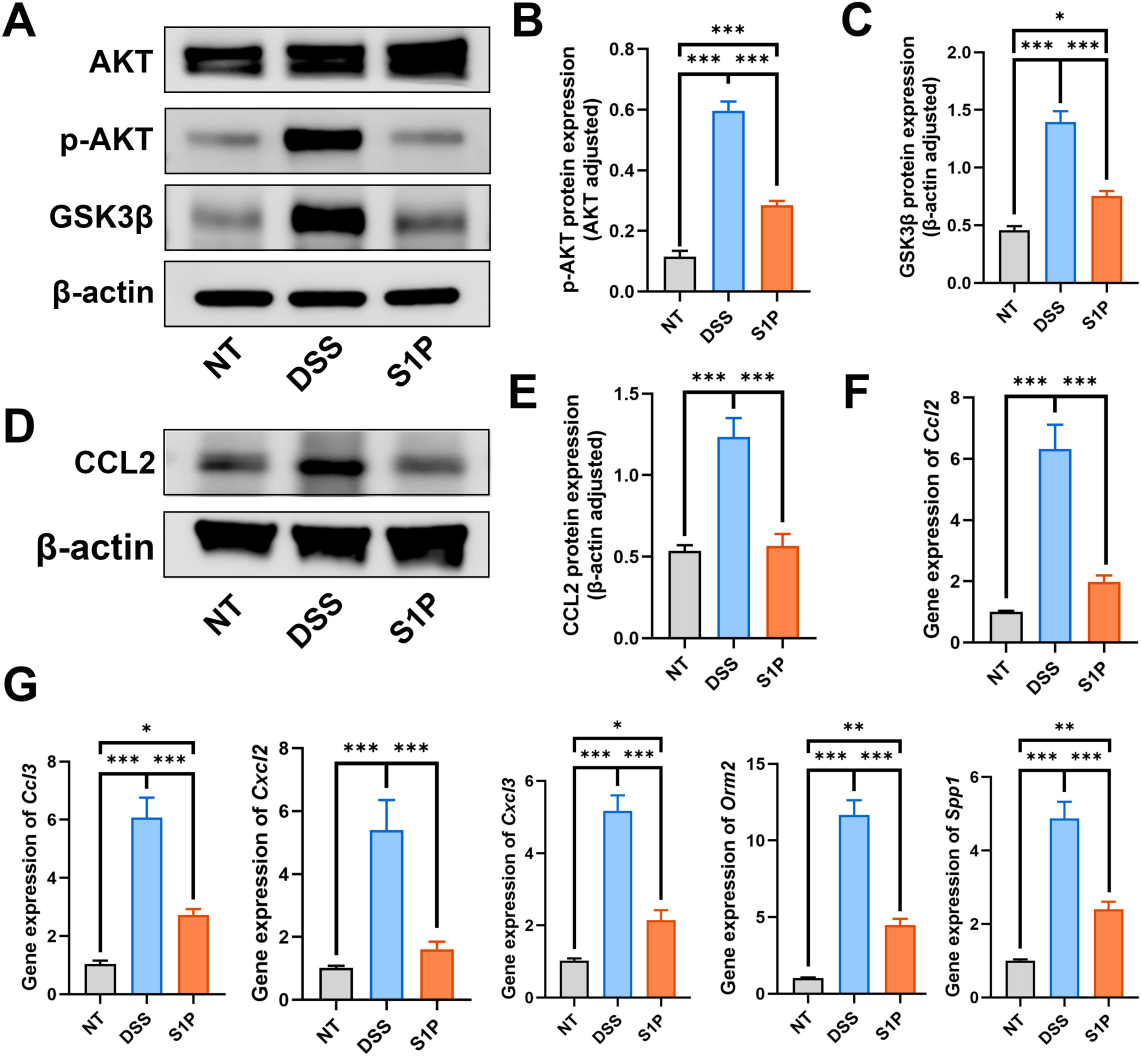
S1P regulated the PI3K-Akt signaling and chemokine signaling pathways. To verify the RNA sequencing results, we detect the protein level of p-AKT/AKT and GSK3b. Results showed that the PI3K-Akt signaling pathway was upregulated in DSS group and downregulated by S1P **(A–C)**. The protein level of CCL2 and the RNA expression of Ccl2 were detected. Our results showed that these markers were increased in DSS group and decreased in S1P group **(D–F)**. The RNA expressipon of Ccl3, Cxcl2, Cxcl3, Spp1 and Orm2 were increased in DSS group and decreased after S1P trestment **(G)**. n=5 for WB and n=3 for PCR examination. *p<0.05, **p<0.01, ***p<0.001.

Our western blotting and PCR findings verified that S1P regulated the PI3K-Akt and chemokine signaling pathways. The ratio of p-Akt/Akt and GSK3β increased in the DSS group and decreased in the S1P group compared with that in the NT group (**Figures 5A–C**). Then, we evaluated changes in C C motif ligand 2 (CCL2) expression, and the protein and mRNA levels of CCL2 were increased in the DSS group and decreased after application of S1P (**Figures 5D–F**). In addition, mRNA levels of *Ccl3*, *Cxcl2*, *Cxcl3*, *Spp1*, and *Orm2*, which are associated with macrophage polarization were significantly upregulated in the DSS group and downregulated in the S1P group compared with those in the control groups (**Figure 5G**).

### 3.6 Polarization of M1 and M2 were suppressed and promoted by S1P, respectively

**Figure 6:**
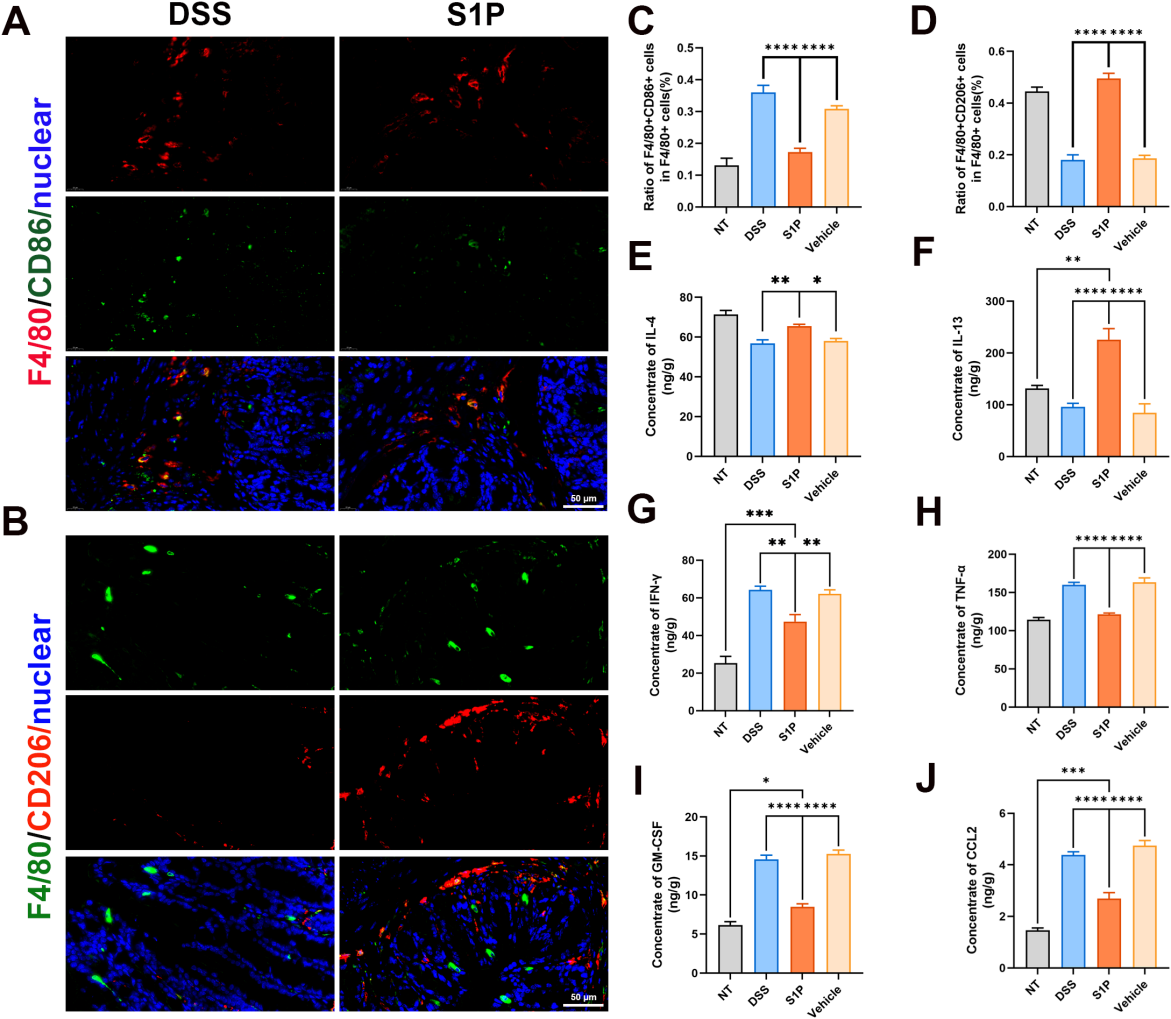
Polarization of M1 and M2 macrophages were respectively suppressed and promoted by S1P. M1 macrophage polarization marker CD86 were detected and the ratio of F4/80+CD86+ cells in F4/80 cells were increased in DSS group compared with NT group, and decreased in S1P group. n=9, ****p<0.0001 **(A, C)**. M2 macrophage polarization marker CD206 were detected and the ratio of F4/80+CD206+ cells in F4/80 cells were decreased in DSS group compared with NT group, and increased in S1P group. n=9, ****p<0.0001 **(B, D)**. The concentration of IL4 and IL13 in intestinal epithelial homogenate decreased in DSS and Vehicle group, and increased in S1P group. n=5, *p<0.05, **p<0.001, ****p<0.0001 **(E, F)**. The concentration of IFN-γ **(G)**, TNF-a **(H)**, GM-CSF **(I)** and CCL2 **(J)** in intestinal epithelial homogenate increased in DSS and Vehicle group, and decreased in S1P group. n=5, *p<0.05, **p<0.001, ***p<0.0001, ****p<0.0001.

We verified that S1P treatment alleviated DSS colitis, and our RNA sequencing results showed downregulated levels of macrophage-associated chemokines in the S1P group compared with those in the DSS group. Therefore, we determined the directions of macrophage polarization and the concentrations of macrophage-associated cytokines. The ratios of F4/80^+^(red) to CD86^+^ (green) macrophages were increased in the DSS group and decreased in the S1P group compared with those in the NT group (**Figures 6A, C**; **Supplementary Figure 3B**). The ratios of F4/80^+^ (green) and CD206^+^ (red) macrophages to F4/80^+^ macrophages were decreased in the DSS group and increased in the S1P group compared with those in the NT group (**Figures 6B, D**; **Supplementary Figure 3A**). We measured the concentrations of macrophage-associated cytokines in intestinal epithelial homogenates. The levels of IL4 and IL13 that promote macrophage M2 polarization were decreased in the DSS and vehicle groups and increased in the S1P group (**Figures 6E, F**). The levels of IFN-γ, TNF-α, GM-CSF, and CCL2, which promote M1 macrophage polarization, were increased in the DSS and vehicle groups and decreased in the S1P group (**Figures 6G–J**).

## 4 Discussion

Our results showed that S1P can alleviate DSS-induced colitis by reducing the HAI and DAI, maintaining intestinal epithelial integrity, and inhibiting apoptosis. We further investigated the mechanisms involved by sequencing RNA isolated from colon samples. The results showed that the PI3K-Akt and chemokine signaling pathways were upregulated in the DSS group compared with those in the S1P group. Our western blotting and PCR findings confirmed the RNA sequencing results. Considering that both the PI3K-Akt and chemokine signaling pathways are associated with macrophage polarization, we analyzed M1 and M2 polarization marker levels. The results showed increased levels of cytokines associated with M1 polarization and a higher ratio of M1 macrophages in the DSS group. The levels of M2 polarization-related cytokines and the ratio of M2 macrophages were higher in the S1P group, indicating that S1P regulated macrophage M1/M2 polarization in mouse models of DSS-induced colitis.

Defective intestinal barrier function can increase intestinal permeability and promote gastrointestinal disease (27). Type I E-cadherin is fundamental for tissue barrier formation, and it plays a vital role in physiological intestinal morphogenesis (28, 29). Occludin, the expression of which is decreased in patients with IBDs, comprises tight junctions in the colonic barrier (30, 31). S1P upregulates the expression of E-cadherin and strengthen intersinal epithelial cell barrier function *in vitro* (32, 33). The deletion of S1P-specific phosphohydrolase isoforms 2 (SGPP2), which catalyze dephosphorylation of S1P to sphingosine could improved DSS-induced mucosal barrier disruption (manifested as increased expression of E-cadherin) (34). The S1PR1 agonist SEW2871 could enhanced barrier function, which resulted from ameliorated tight junction (occludin and ZO-1) (35). In the present study, we found that S1P maintained intestinal barrier function via upregulating E-cadherin and occludin expression. All these results indicate that the S1P-S1PR pathway protects the epithelial barrier by strengthening the cell junctions.

Increased rates of apoptosis in colonic tissues, especially at crypts, play vital roles in the pathogenesis of IBD (36–38). Furthermore, the level of intestinal epithelial cell apoptosis is associated with disease severity in patients with active ulcerative colitis (UC), indicated by apoptotic bodies in colonic biopsies (39). Similar to its effects on the intestinal barrier, SEW2871 inhibits epithelial cell apoptosis in IL-10-knockout mice (35). We detected TUNEL-positive cell abundance and cleaved caspase 3 and Bax protein expression in colonic tissues, which revealed that S1P significantly decreased DSS-induced apoptosis and alleviated the severity of colitis.

We sequenced RNA to investigate the mechanism through which S1P alleviated DSS-induced colitis in model mice. Differences in gene expression between the DSS and S1P groups confirmed that S1P exerted a protective effect against colitis induced by DSS in the models. Glutathione peroxidase 2 (Gpx2) reduces p53-dependent oxidative stress, and its expression was downregulated in the DSS group compared with that in the S1P group (40). Gene expression of the highly selective calcium channel, Transient Receptor Potential Vanilloid Subfamily Member 6 (TRPV6), which is significantly higher in patients with active UC, was notably lower in the DSS than in the S1P group (41). The expression of chemokine (C-C motif) ligand 3 (Ccl3) and chemokine (C-X-C motif) ligand 2 (Cxcl2), which are associated with inflammation, was upregulated in the DSS group compared with that in the S1P group (42, 43). Overall, the RNA sequencing results revealed that the expression of genes associated with oxidative stress and inflammation was downregulated in the S1P group.

The expression of secreted phosphoprotein 1 (SPP1; Osteopontin), which dephosphorylates S1P to sphingosine, was upregulated in the DSS group compared with that in the S1P group. The PPI analysis revealed a positive correlation between SPP1 expression and the expression of the chemokine signaling pathway markers Ccl3 and Cxcl2 (44). These data indicated that decreased S1P levels are associated with activation of the chemokine signaling pathway.

RNA sequencing analysis indicated that therapeutic effects of S1P may involve suppression of focal adhesion and ECM-receptor interaction pathways, which were downregulated versus the DSS group. In five pediatric UC patients, RNA sequencing from intestinal mucosal samples found that DGEs enriched in ECM-receptor interaction pathways (45). ECM-receptor interaction were involved in the development of colitis-associated colorectal cancer (46). In addition, the focal adhesion complex (FAC) has been implicated in the pathogenesis of IBD (47). These results demonstrate that focal adhesion and ECM-receptor interaction pathways contribute significantly to IBD pathogenesis and treatment, warranting further investigation.

PI3K/Akt and chemokine signaling pathways were upregulated in the DSS group compared with those in the S1P group. Activation of the PI3K/Akt pathway induces NF-κB signaling, activates inflammatory cells, and promotes inflammatory cytokine release, all of which become overactive in colitis (48). A large number of p-Akt positive cells were present in the intestinal mucosal biopsy tissues of UC patients (49). Application of PI3K inhibitors could significantly improved the histological scores in the mouse colitis models (48, 49). Astragaloside IV treatment attenuated inflammation and restored intestinal epithelial integrity by suppressing PI3K/AKT signaling (38). Vitexin can attenuate neuropathic pain and increase serum S1P levels, subsequently inhibiting the PI3K-Akt signaling pathway and regulating astrocyte polarization (50). In the present study, our group showed that the upregulation of oxidative stress, inflammation marker levels, and the PI3K-Akt signaling pathway were associated with macrophage M1 polarization in the DSS group. Furthermore, S1P significantly downregulated the PI3K/Akt and chemokine signaling pathways. Thus, we subsequently assessed M1 and M2 macrophage polarization *in vivo*.

Macrophages reside in many tissues as they play various roles in cytokine secretion, inflammation, tissue repair, and angiogenesis (51, 52). We found that increased levels of the M1 polarization marker CD86 and related chemotactic factors in the DSS group were decreased following S1P treatment. S1P increased the levels of the M2 polarization marker CD206 and decreased related chemotactic factors, respectively. M1 macrophages are mainly involved in proinflammatory responses; they produce proinflammatory cytokines and promote colitis (53, 54), whereas M2 macrophages are mainly involved in anti-inflammatory responses and promote the repair of damaged tissues (53, 54). The increased and decreased numbers of M2 and M1 macrophages, respectively, in the S1P group suggested the alleviation of DSS-induced colitis.

However, the mechanism by which S1P regulates the PI3K/Akt signaling pathway and the polarization of macrophages remains unclear and requires further investigation. Overall, our findings indicated that S1P alleviated DSS-induced colitis in mice by promoting and suppressing macrophage M2 and M1 polarization, respectively, and by regulating the PI3K/Akt signaling pathway.

## Data Availability

The expression data have been deposited in the National Center for Biotechnology Information under the accession number PRJNA1243919 (https://www.ncbi.nlm.nih.gov/bioproject/PRJNA1243919).
