# Supplementary material for: Sphingosine-1-phosphate alleviates colitis by regulating macrophage polarization and PI3k-Akt signaling
Source: Front Immunol. 2025 Jul 21;16:1622094. doi: 10.3389/fimmu.2025.1622094 (PMC12318956; doi:10.3389/fimmu.2025.1622094)

**Supplementary Table 1 Primer Sequences for qRT-PCR assays**

| Genes | Primers-Forward | Primers-Reverse |
| --- | --- | --- |
| *Actb* | TATGCTCTCCCTCACGCCATCC | GTCACGCACGATTTCCCTCTCAG |
| *Spp1* | AGAGCGGTGAGTCTAAGGAGTCC | TGGCTGCCCTTTCCGTTGTTG |
| *Ccl3* | CCACCACTGCCCTTGCTGTTC | GCGTGGAATCTTCCGGCTGTAG |
| *Cxcl3* | CACTGGTCCTGCTGCTGCTG | CGTCACCGTCAAGCTCTGGATG |
| *Cxcl2* | CACTGGTCCTGCTGCTGCTG | CACTGGTCCTGCTGCTGCTG |
| *Orm2* | GCTGCGGGAAGTATTCCAGAAGG | GCTGCTGCTTCTCCTGCTGAC |
| *Ccl2* | CCACTCACCTGCTGCTACTCATTC | CTTCTTTGGGACACCTGCTGCTG |

Supplementary Figure 1


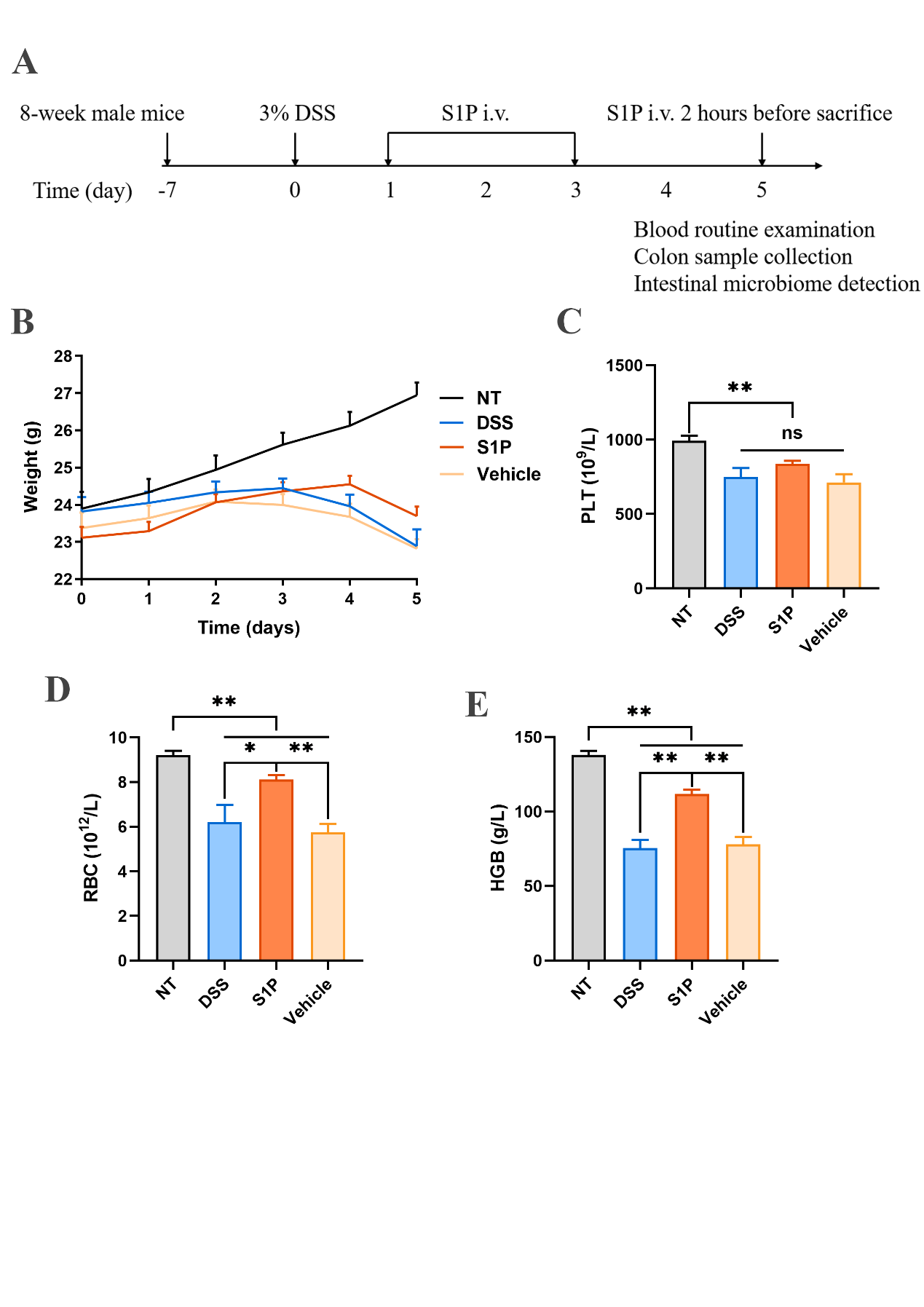


Supplementary Figure 2


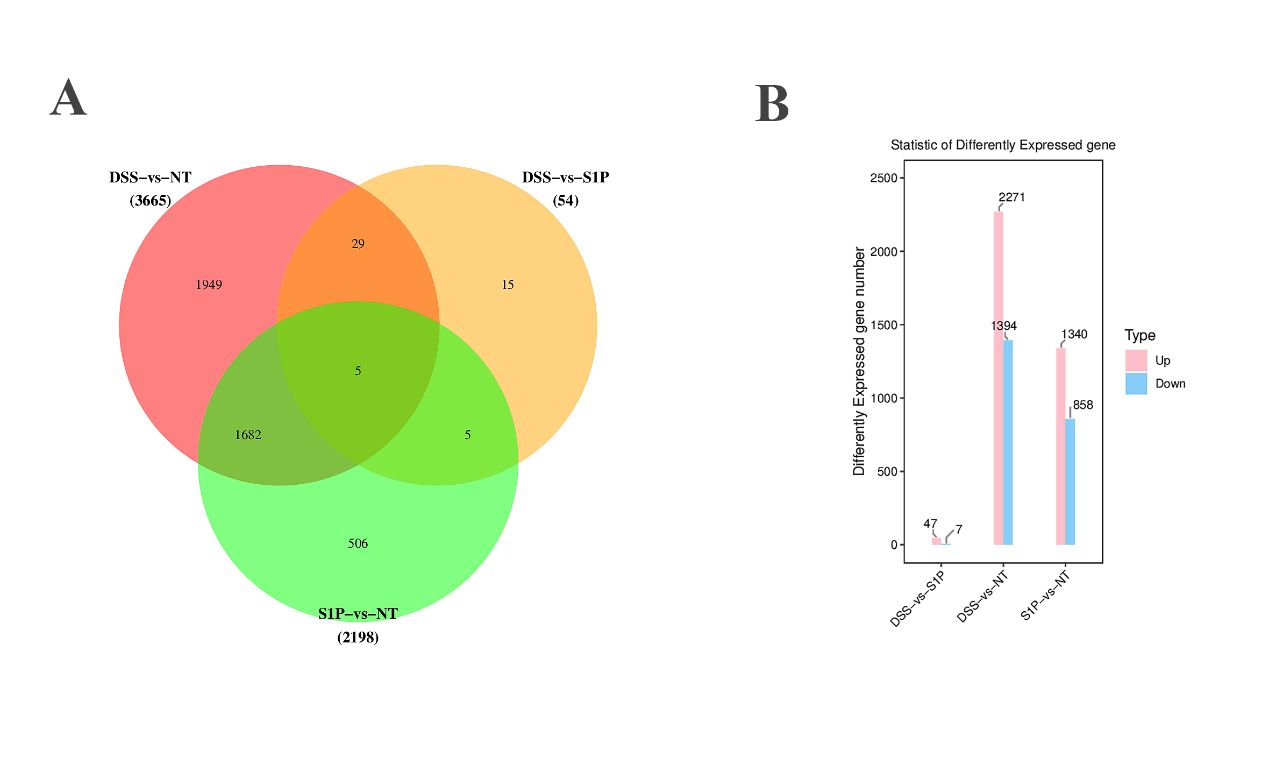


Supplementary Figure 3


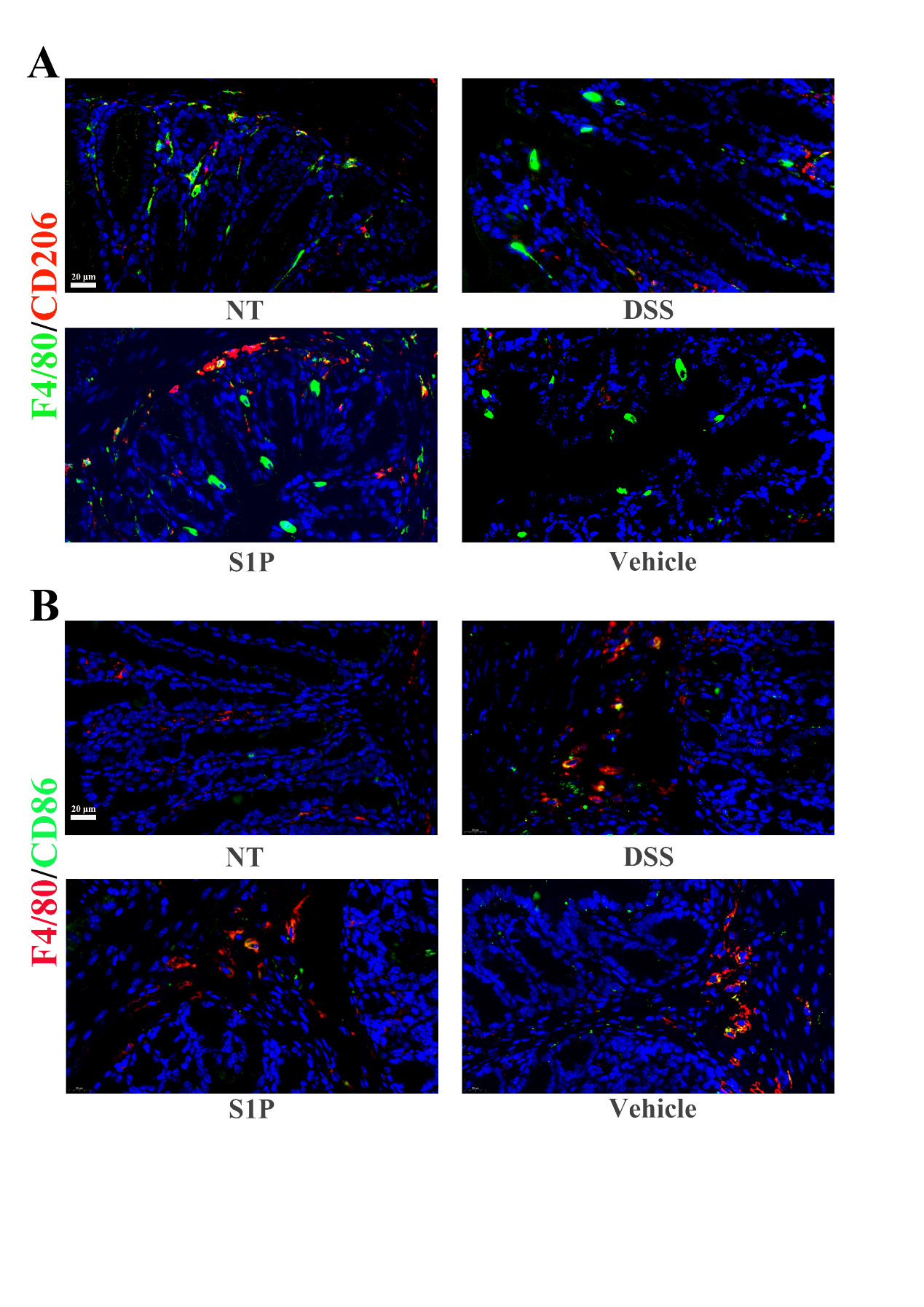

Supplement: Supplementary file 1 [file Table1.docx]
